# The application of focused assessment with sonography for trauma in resource-limited settings: a scoping review

**DOI:** 10.3389/fpubh.2026.1774992

**Published:** 2026-03-16

**Authors:** Yuhan Song, Shuang Han, Wen Xu

**Affiliations:** 1 Department of Nursing, School of Health, China Three Gorges University, Yichang, China; 2 Emergency Department, The First Clinical Medical College, Yichang Central People’s Hospital, China Three Gorges University, Yichang, China

**Keywords:** focused assessment with sonography for trauma, resource-limited, scope review, sonography, ultrasound

## Abstract

**Background:**

Trauma represents a leading cause of mortality and disability in resource-limited settings. However, access to advanced imaging modalities is severely constrained, limiting timely and accurate diagnosis as well as clinical decision-making. Focused assessment with sonography for trauma (FAST) offers a portable, non-ionizing, and cost-effective bedside imaging solution with significant potential for clinical application. It enables rapid identification of life-threatening hemoperitoneum or hemothorax, thereby guiding immediate surgical intervention or preventing unnecessary patient transfers. As such, FAST contributes to improved triage, reduced decision-making time, and enhanced efficiency in trauma care delivery within these contexts. Nevertheless, there remains a lack of comprehensive synthesis regarding its implementation models, effectiveness, and associated challenges in low-resource environments.

**Methods:**

Adhering to the PRISMA 2020 guidelines, a systematic literature search was conducted across six databases—PubMed, Embase, Cochrane Library, Scopus, Web of Science, and SinoMed—up to October 11, 2025. Study screening, quality assessment, and data extraction were performed independently, resulting in the inclusion of 29 eligible studies.

**Results:**

The synthesis of 29 included studies demonstrates that FAST is a highly effective tool in resource-limited settings. It exhibits high specificity (94%–100%) in detecting free intraperitoneal fluid, supporting reliable clinical decision-making. FAST has been successfully integrated into diverse healthcare contexts, including pre-hospital emergency response and primary care clinics. A critical enabler of its implementation is task-shifting, with nurses, general practitioners, and other non-specialist healthcare providers serving as primary operators. Nonetheless, persistent challenges include limited equipment availability and difficulties in maintaining operator competency, underscoring the need for innovative solutions such as remote tele-guidance, cascade training programs, and the development of local training capacity.

**Conclusion:**

As a rapid, portable, and cost-efficient diagnostic modality, FAST plays a vital role in strengthening trauma care systems in resource-limited settings. Despite existing limitations, its sustainable scale-up depends on the integration of technological access, robust training frameworks, and supportive health policies. Such a multifaceted approach is essential to improving survival rates and long-term outcomes for trauma patients in these regions and advancing global trauma care equity.

**Systematic review registration:**

https://doi.org/10.17605/OSF.IO/2T7BK.

## 1 Introduction

Trauma is a leading global cause of death and disability (1). According to the World Health Organization (WHO), unintentional injuries and violence result in approximately 4.4 million fatalities annually. Young adults aged 15–44 are disproportionately affected, imposing a significant societal and economic burden. Notably, over 90% of these trauma-related deaths occur in low- and middle-income countries (LMICs) (2). In these resource-limited settings, fragile public health infrastructure, scarcity of medical resources, and underdeveloped emergency response systems place immense strain on healthcare capacities (3). Consequently, patients often face poor prognoses due to delayed or absent diagnostic imaging and treatment, while frequent trauma incidents drive up readmission rates, surgical demands, and mortality-associated costs, presenting a critical challenge to trauma management.

Survival in trauma is critically dependent on timely and efficient diagnosis (4, 5). Computed tomography (CT), the current diagnostic gold standard, offers high sensitivity and specificity but is hindered by its high cost, radiation exposure, and requirements for patient stability and transport—factors that severely limit its feasibility in resource-limited environments (6–8). Magnetic resonance imaging (MRI) faces similar constraints regarding accessibility and speed. In contrast, point-of-care ultrasound (POCUS) provides a portable, radiation-free, low-cost alternative that can be performed at the bedside. However, comprehensive ultrasonography can be time-consuming, potentially delaying critical decisions in emergencies. Therefore, in trauma care settings with limited resources, a rapid, reliable, and accessible bedside tool is essential to effectively support clinical decision-making, thereby enhancing the timeliness and overall quality of trauma care in these regions.

To address this need, the FAST was developed as a rapid, goal-directed bedside tool. First systematically described and promoted by Rozycki et al. in the 1990s, Its core objective is to rapidly screen for the presence of free fluid in the thoracic and abdominal cavities following trauma (indicating bleeding), rather than to replace advanced imaging studies such as CT scans. The FAST exam was designed to quickly detect free intraperitoneal or pericardial fluid (suggestive of hemorrhage) using standard views (sub-xiphoid, right upper quadrant, left upper quadrant, and pelvic) (9, 10). In high-resource settings, FAST has become a standardized component of advanced trauma life support protocols and a routine screening tool in emergency departments, with well-defined operator competencies integrated into regional trauma networks (11, 12).

Conversely, the promotion and application of FAST in settings where resources are limited poses a distinct set of challenges. In these areas, where advanced imaging resources such as CT are extremely scarce, FAST is a viable tool for initial assessment and triage due to its simple, rapid, non-invasive, low-cost, and portable nature (13, 14). And is particularly suited for the initial screening process of trauma patients in pre-hospital, primary, and remote areas. It’s simple and easy-to-learn feature also facilitates the rapid training of FAST operation teams through short-term training, thus realizing rapid promotion and application in resource-limited areas.

However, the current application status, effectiveness verification, and promotion model of FAST in resource-limited regions facing the most severe trauma challenges have not been comprehensively summarized. Most of the existing evidence originates from high-resource environments or focuses on the general trauma population. Systematic evidence specifically addressing the unique context of resource-limited regions—such as equipment shortages, reliance on non-physician operators, and underdeveloped training systems—remains scarce. Furthermore, many related studies are limited by small sample sizes and single-center designs, which restricts the generalizability of their conclusions.

Therefore, this study aims to systematically review the literature on FAST application in resource-limited regions to clarify its application models, diagnostic performance, clinical utility, effective training strategies, as well as the core barriers and facilitators in the implementation process. The goal is to provide an evidence-based foundation for optimizing trauma care practices, personnel training, and health policy formulation in these regions. This enhances the timeliness and effectiveness of trauma care in the region, reduces the incidence of trauma-related death and disability, and reduces the socioeconomic burden.

## 2 Methods

This scope review is based on the Arksey and O’Malley framework (15, 16); (1) to determine the research questions; (2) to identify the relevant studies; (3) Research selection; (4) Data collection; (5) Organize, summarize and report the results. This framework provides an excellent methodological foundation for it. The research results will follow PRISMA-ScR (Preferred Reporting Items for Systematic Reviews and Meta-Analyses extension for Scoping Reviews) Report in accordance with the PRISMA-ScR guidelines (17, 18). The scheme in the open scientific framework (OSF) prospective registration^1^.

### 2.1 Identifying the research question

The objective of this study is to systematically review the current application status of FAST in resource-limited settings. This review aims to address specific research questions: (1) In which clinical environments is FAST utilized, and who primarily operates it? (2) What are the sensitivity and specificity rates associated with FAST? (3) What clinical advantages does FAST offer? (4) What effective training models exist for implementing FAST? (5) What are the primary challenges encountered in the implementation of FAST, and what factors contribute to its successful adoption?

### 2.2 Identifying relevant studies

With the assistance of an experienced research librarian, we conducted a comprehensive search across six databases: PubMed, Embase, Cochrane Library, Scopus, Web of Science, and SinoMed. All databases were accessed from their inception up to October 11, 2025. To improve the accuracy and reproducibility of the search, the language of this search was limited to English. PubMed served as the primary database for our search strategy.

### 2.3 Study selection

This study aims to comprehensively integrate research on the application of FAST in resource-limited settings. Accordingly, we established the inclusion and exclusion criteria presented in Table 1 based on clearly identifiable populations, concepts, and contexts (PCC).

**Table 1:** Inclusion and exclusion criteria.

Elements of PCC	Inclusion criteria	Exclusion criteria
Population	Patients of all ages (children and adults) who visited the hospital due to trauma are mainly focused on the actual trauma treatment in resource-limited areas, and the personnel of various groups (not limited to medical personnel) who participate in the key assessment of trauma ultrasound are focused on the research of improving the trauma ultrasound skills of this group through simulation training.	Patients with medical conditions such as stroke, myocardial infarction, etc.
Concept	The focus is on the application of FAST, including but not limited to the operation process, accuracy, diagnostic performance, impact on clinical decision-making, examination time, operator training methods, and integration time in the treatment process	The study focused on other forms of ultrasound (e.g., whole-body ultrasound, cardiac ultrasound), and non-core components of FAST, including the extended focused assessment of trauma ultrasound (Efast), only mentioned “ultrasound was used” but did not explicitly refer to FAST or describe its application details.
Research background	Geographical and economic aspects: LMICs (based on the World Bank’s income stratification standards) and remote or rural areas in high-income countries; Medical resource level: it refers to environments with limited medical facilities, equipment or human resources, such as war zones, disaster sites, areas with unstable power supply, medical institutions lacking advanced imaging equipment like CT or specialized trauma surgeons. Medical scene level: it includes pre-hospital emergency care, primary care clinics, regional or county-level hospitals and field hospitals, etc.	Studies conducted in well-resourcated tertiary grade A hospitals or top trauma centers, and whose conclusions are highly dependent on the resources of these centers (such as readily available CT and interventional radiology departments)

### 2.4 Data collection

The review was designed to include a wide range of relevant evidence, so the types of studies were not limited. The main inclusion criteria were original studies that evaluated or described the application of FAST. The designs covered randomized controlled trials, cohort studies, case–control studies, cross-sectional studies, before-after control studies, and case series (defined in this study as research reporting at least three similar cases consecutively). Additionally, case reports (defined as studies detailing one or two cases), qualitative studies, mixed methods studies, descriptive studies and project reports were also included. According to the study objectives, scoping reviews that fit the topic will also be included, mainly for the drawing of evidence maps, from which the original study data will not be extracted secondary.

### 2.5 Collation, summary, and reporting of the results

The following data were extracted from each included study: author, publication year, country, type of setting, study design, subjects and population, FAST operator, and main study focus covering: (1) application overview, (2) diagnostic performance, (3) clinical advantages, (4) training modalities, and (5) barriers and facilitators to implementation. All data were extracted and managed using standardized forms.

**Figure 1:**
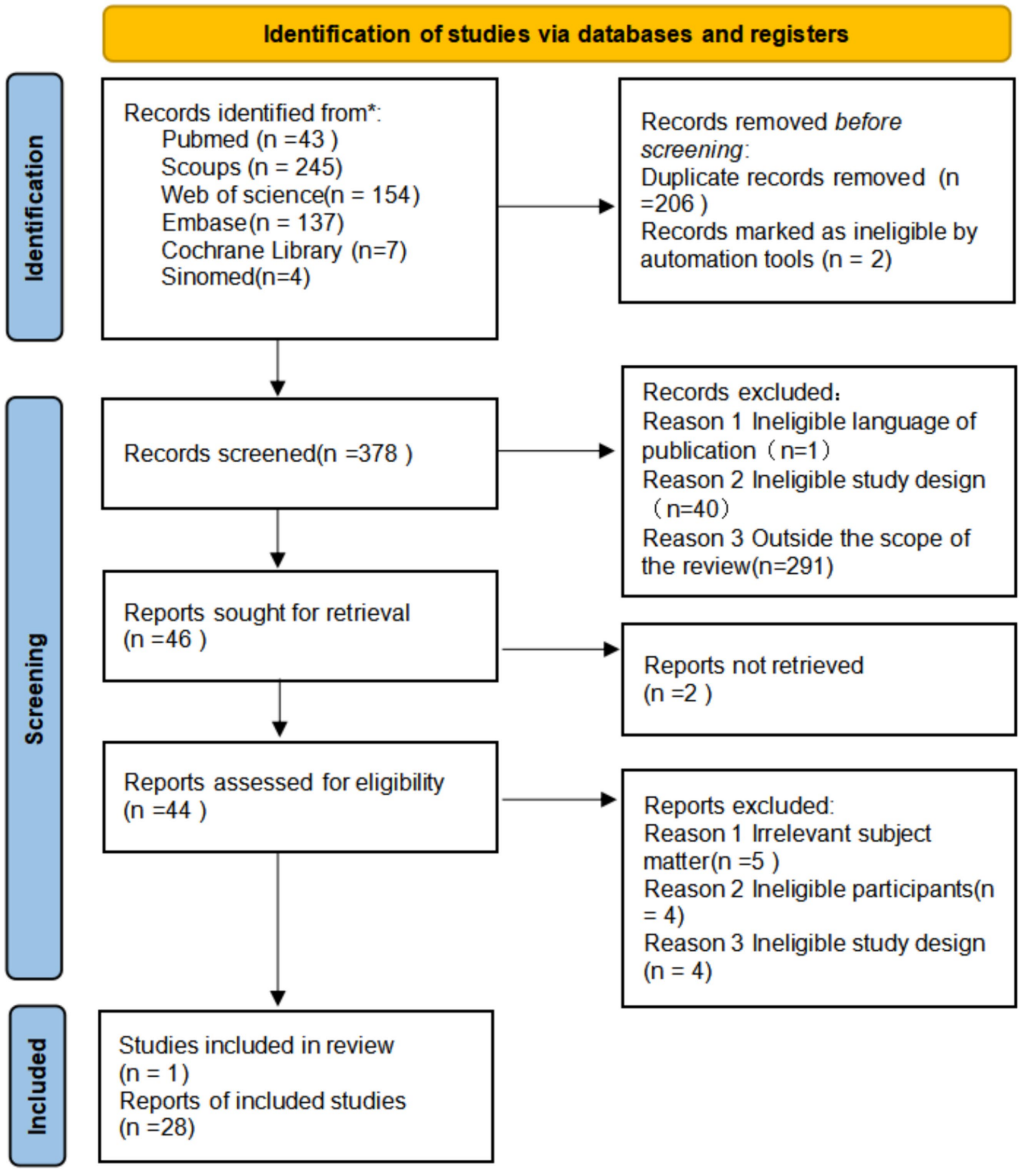
Scoping review flowchart.

The literature screening procedure is detailed in Figure 1; a total of 590 records were obtained in the initial search. Zotero software was used to eliminate 208 duplicate articles, and the remaining 378 articles entered the screening stage. After primary screening by title and abstract, 334 articles were excluded. Subsequently, 44 articles were evaluated for full text, and 2 of them were excluded because they could not obtain full text. Finally, 29 studies met the inclusion criteria and entered the stage of data extraction and analysis. Study results were reported in accordance with PRISMA-ScR guidelines.

## 3 Result

### 3.1 Basic characteristics of the included studies

A total of 29 eligible studies were included in this study. Table 2 presents the basic characteristics of the included literature. These studies were published between 2010 and 2025 and covered multiple resource-limited, low- and middle-income countries, including South Africa, Pakistan, Uganda, Rwanda, India, and Iraq. The types of research designs are diverse, mainly including prospective and retrospective studies (*n* = 13), cross-sectional surveys (*n* = 7), case series (*n* = 1), case reports (*n* = 3), pre- and post-intervention controlled studies (*n* = 2), Delphi studies (*n* = 1), and scope reviews (*n* = 2). The sample size of the research subjects ranged from case reports to over 4,000 trauma patients. All studies have focused on the application of FAST in resource-limited environments. The research priorities can be summarized into five main aspects: (1) application breadth and model, (2) diagnostic performance, (3) clinical advantages, (4) training model, (5) obstacles and promoting factors in the implementation process.

**Table 2:** Basic characteristics of the included literature.

Author	Year	Country	Environmental type	Study design	Population	Operator	Research focus
Nixon et al. (20)	2019	New Zealand	Rural hospital	Prospective study	Blunt trauma in adults	General medical practitioner	①②
Moher et al. (27)	2023	Malawi	Regional hospital	Retrospective study	Pediatric emergency patients	Pediatric emergency physician	①③
Gillani et al. (23)	2020	Pakistan	Trauma center	Cross-sectional study	Pregnant women with trauma	Undefined	②③
Ahmed et al. (37)	2017	Bangladesh	Teaching hospital	Comparison before and after the intervention	Trainees (simulation)	ICU/emergency physician	④
Lolar (28)	2022	America	Training item	Cross-sectional study	Trainees (students)	Student	④⑤
Eisa et al. (43)	2018	Egypt	Trauma center	Retrospective study	Patients with pelvic fractures	Undefined	②③
Terry et al. (26)	2019	Uganda	Rural areas	Prospective study	Trainees (non-physicians)	Non-physician clinical doctor	②④
Henwood et al. (40)	2013	Rwanda	Regional hospital	Prospective study	Physicians	Physician	①③④
Shah et al. (44)	2015	44 countries including Uganda, Rwanda, Haiti, etc.	Low- and middle-income areas	Cross-sectional study	Medical staff	Doctor/nurse	①⑤
Wadaja et al. (46)	2025	Ethiopia	Regional hospital	Case report	Penetrating trauma (puncture)	Undefined	②③
Omari et al. (35)	2013	Jordan	Teaching hospital	Retrospective study	Blunt trauma in adults	Undefined	①②③
Al-Sindy et al. (21)	2018	Iraq	Theater command hospital	Case report	Blunt trauma in adults		①②③
Nathani et al. (39)	2024	India, Belarus, Azerbaijan, etc.	Low- and middle-income areas	Scope review	Trauma patients	Doctor/nurse/student	①④⑤
Kotagal et al. (49)	2015	America	Training item	Prospective study	Trainees (physicians)	House-surgeon	④
Muhammad et al. (34)	2018	Pakistan	Regional hospital	Cross-sectional study	Blunt trauma in adults	Radiology resident	②④
Shaffer et al. (41)	2017	Tanzania	Referral hospital	Prospective study	Trauma patients	Registered medical practitioner	④
Mohamadi and Ghasemi-Rad (31)	2012	Iran	University hospital	Retrospective study	Blunt trauma in adults	Emergency physician	②
Smith et al. (38)	2010	South Africa	Rural hospital	Prospective study	Patients with trauma to the chest and abdomen	Emergency physician	②
Adams et al. (25)	2024	Undefined	Military austere environments	Delphi research	Patients with abdominal bleeding	Assistant surgeon	①③
Aspler et al. (42)	2022	Ethiopia	Teaching hospital	Cross-sectional study	Trainees (resident physicians)	Emergency resident doctor	④⑤
Cioè-Peña et al. (24)	2016	Salvador	National hospital	Prospective study	Trauma patients	Surgeon/nurse	①③⑤
Rupp et al. (33)	2017	Iraq	Conflict zone	Case series	Wounded civilians	Undefined	①③
Soni et al. (19)	2019	India	Trauma center	Retrospective study	Patients with mixed trauma	Undefined	①③
Abdel Hamid et al. (22)	2024	Egypt	University hospital	Retrospective study	Blunt trauma in adults	Emergency physician	②③
Munihire et al. (45)	2023	Uganda	Teaching hospital	Case report	Pelvic fracture (unstable)	Undefined	③⑤
Nelson et al. (50)	2010	Undefined	War and disaster environments	Scope review	Trauma and emergency patients requiring assessment in remote or resource-limited environments	Diversity (doctors, nurses, emergency responders, military doctors.)	①③④⑤
Wanjiku et al. (29)	2018	Kenya	Rural hospital	Cross-sectional study	Trainees (rural workers)	Rural medical workers	④⑤
Tullavardhana and Rookkachart (30)	2017	Thailand	Medical college	Comparison before and after the intervention	Trainees (medical students)	Medical student	④
Montazer et al. (32)	2016	Iran	Teaching hospital	Cross-sectional study	Blunt trauma in adults	Emergency resident doctor	②

The main research focuses are shown in Tables 3–7 below (① Application breadth and model; ② Diagnostic performance; ③ Clinical advantages; ④ Training model; ⑤ Obstacles and promoting factors).

### 3.2 The application breadth and integration model of FAST

FAST has demonstrated extensive applicability and flexible integration models in various resource-limited environments (see Table 3). For instance, a research report from a primary trauma center in India indicates that the application rate of FAST is as high as 99.2% (19). In a multitude of scenarios, FAST has become a pivotal preliminary assessment tool for trauma patients, and in some cases, it is the sole imaging modality employed (20–22). In low- and middle-income areas or extreme environments with slightly better facilities, such as rural areas, war zones or primary medical institutions, the utilisation rate of FAST is extremely high. The concept of “fast” is integrated into a variety of clinical scenarios, including pre-hospital emergency care, initial assessment in the emergency department, trauma resuscitation processes, and the assessment of specific trauma groups, such as pregnant women (23–25). It is worthy of note that certain studies have reported the creative application of FAST in non-traumatic indications (such as the assessment of oedema in children, respiratory distress, and suspected infection in non-traumatic children), and these studies have demonstrated a relatively high positive detection rate. The potential for application of this technology may exceed that of the traditional trauma field (23). The medical personnel hail from a variety of professional backgrounds, including emergency medicine, surgery, radiology, general practice, and non-physician clinical roles such as emergency care staff. The workforce also comprises medical students and residents in the training stage (26–30).

**Table 3:** Overview of the application of FAST in resource-limited areas.

Application dimension	Main discovery	Literature
Operator type	Doctors (emergency physicians, surgeons, general practitioners)	(20, 21, 24, 26–30, 34, 38–44, 49, 50)
	Non-physician clinicians (nurses, paramedics)	
Clinical scene	Emergency Department	(19–21, 23–25, 27, 39, 50)
	Prehospital care	
	Primary care clinics	
	Field hospital	
Integration mode	Initial assessment of trauma patients	(20, 21, 24, 27, 38, 39, 50)
	Operator in-service training for trauma patient assessment	
	Participate in trauma resuscitation process guidance and triage	
	The primary or only imaging tool in areas lacking CT	

### 3.3 Diagnostic performance and clinical utility of FAST

#### 3.3.1 Diagnostic performance

Multiple studies have reported the diagnostic performance of FAST in resource-limited settings (see Table 4). It usually shows high specificity (94%–100%) for the detection of free intra-abdominal fluid, but its sensitivity varies widely (73%–88%), especially in isolated cavity-organ injury (38.5%) (31, 32). The strategy of repeated ultrasound examination can be used in cavitary organs with low diagnostic sensitivity. In order to enhance the detection rate, it is possible to increase the sensitivity of gastrointestinal injury to 85.2% (31). Despite the scarcity of resources in combat zones, a series of case reports have demonstrated the efficacy of a novel approach to the detection of occult injuries (33). This method employs high-frequency linear probes for systematic scanning and incorporates advanced versions of traditional FAST technology. The utilization of these techniques has yielded successful detection outcomes, even in situations where conventional methods might have failed to identify such injuries. The results of FAST showed a high degree of agreement (Kappa = 0.84) between different seniority operators (junior and senior radiology residents), indicating that FAST is a relatively simple, easy to learn, and standardized trauma assessment tool. Even inexperienced doctors with standardized training can obtain results as reliable as those of senior doctors. This is especially important in health centers in poor, limited, or remote areas (34).

**Table 4:** Diagnostic performance of FAST in resource-limited areas.

Index of performance	Summary results	Literature
Sensitivity	Large variation (73–88%); Low damage to isolated cavity organs (38.5%)	(31, 32, 38, 43)
Specificity	High and stable (94–100%)	(31, 32, 35, 43)
Operator consistency	High (Kappa = 0.84), indicating that it is easy to standardize and can be mastered by people with different experiences	(34)
Technical improvements	Repeated ultrasound and systematic scanning with high-frequency linear probes can significantly improve the detection of occult injuries such as gastrointestinal injuries	(31, 33)

#### 3.3.2 Clinical advantages of FAST

The clinical application value of FAST is significant. This approach has been demonstrated to reduce the evaluation time of trauma patients significantly, while concomitantly enhancing the efficacy of the emergency decision-making process. Its value is reflected in four aspects: guiding clinical treatment, predicting patient outcome, improving diagnostic accuracy and optimizing resource utilisation. In hemodynamically unstable patients, a positive FAST result can strongly support the decision for urgent surgical intervention (21, 35), in patients who are relatively hemodynamically stable, a positive FAST is an important warning sign that suggests the need for a higher level of monitoring, serial evaluation, or consideration of further imaging/surgical exploration (36), negative results, on the other hand, can support initial conservative management under close observation or avoid unnecessary immediate referrals, thereby optimizing triage efficiency in resource-limited settings (20), thereby facilitating de-escalation and rapid diversion of treatment, thus enhancing the efficiency of the trauma treatment process. In addition, a large retrospective cohort study confirmed that FAST was an independent predictor of in-hospital mortality in trauma patients (OR = 2.54) (26). In practice, most physicians reported that FAST significantly increased their diagnostic confidence (20, 37). In areas with limited CT resources, FAST, as a rapid bedside screening tool, is viewed as an important part of the diagnostic process rather than an endpoint and is effective in capturing critical condition changes (38, 39), and its findings need to be combined with the patient’s mechanism of trauma, vital signs, and trauma-related scores in order to avoid over intervention (22) (see Table 5).

**Table 5:** Clinical advantages of FAST in resource-limited settings.

Project	Specific effects	Literature
Guidance for decision making	FAST test results drive critical clinical decisions	(20, 26, 33, 35, 38, 39, 43)
Prediction of outcome	Positive FAST was an independent predictor of in-hospital mortality (OR = 2.54)	(26)
Confidence and diagnostic certainty	After performing FAST, their confidence in judging the condition was significantly enhanced	(20, 37)
Resource optimization	Quickly allocate critical resources	(20, 38, 39)

### 3.4 Training mode of FAST

In resource-limited Settings, FAST has varied training modalities and a wide range of participants (see Table 6). The main training models included short (1-2 d) intensive workshops, continuing education with distance quality feedback, and student interest groups integrated into the medical school curriculum (30, 37, 40). Effective training models frequently incorporate a combination of theoretical teaching, image interpretation, simulation training (utilizing simulated patients or homemade low-cost models), and supervised clinical practice (28, 30, 41). The training target not only included doctors, but also successfully extended to non-physician clinical personnel, such as emergency nursing and rural nursing workers, achieving effective task transfer (40). The common training content focused on the acquisition and interpretation of FAST standard sections, and the competency assessment included written examination, objective structured clinical examination, image quality score and confidence questionnaire survey (42). The design of a structured curriculum, the implementation of continuous supervision and feedback mechanisms, and the training of local lecturers are all closely related to the success and orderly progress of the training (24). However, skill maintenance is a major challenge, as both studies indicated that a single training session is often not enough to enable participants to acquire and maintain a skilled skill for a long time. It is a common issue among students that they do not have sufficient opportunities to practice. Although training can significantly improve operator confidence (28, 34), lack of sustained practice can lead to skill deterioration (31).

**Table 6:** Training patterns of FAST in resource-limited areas.

Dimensions of training mode	Concrete content	Literature
Training objects	Doctor (emergency, surgery, general practice)	(24, 26, 28–30, 38–43, 49)
	Non-physicians (nurses, emergency care workers, clinical officers)	
	Trainees (medical students, residents, physician assistants)	
Training pattern	Short training workshop (1-2 d)	(21, 24, 28–30, 38–43)
	Comprehensive training program (months): theory, simulation and supervised practice	
	Distance education and quality feedback	
	Study group	
Core teaching methods	Theory teaching and image interpretation	(24, 28–30, 38, 39, 41–43, 49)
	Simulated training	
	Supervised clinical practice	
Capacity assessment methods	Written test (knowledge test)	(28–30, 32, 38, 39, 41, 43)
	Objective structured clinical examination (skill assessment)	
	Image quality score	
	Self-confidence questionnaire survey	
Key elements of success	Structured curriculum design	(24, 28, 29, 39, 40, 42, 43, 49)
	Training of local lecturers	
	Continuous supervision and feedback mechanism	
	The training content was closely related to local clinical needs	

### 3.5 Barriers and key enablers of FAST implementation in resource-limited areas

There are many challenges in promoting FAST in resource-limited areas. The successful implementation of FAST depends not only on the technology itself, but also on the comprehensive impact of local resource conditions, manpower structure, training system and policy environment (24, 27, 34, 42–44). A number of issues have been identified, including a shortage of equipment, difficulties in maintenance, and high costs in materials and equipment (29, 30, 33, 38, 39, 45). Infrastructure shortage of power, network and basic consumables (29, 41, 45, 46); Lack of standardized curriculum and clinical process integration in system and policy (32, 39). Some studies have also shown a number of key promoting factors, which provide an important basis for formulating effective implementation strategies in the future. The specific facilitating factors are shown in Table 7.

**Table 7:** Major barriers and facilitators of FAST in resource-limited areas.

Category	Key barriers	Key enablers
Manpower resource	Local ultrasound specialists are scarce	Shift of task
	Brain drain	Training of local lecturers
Equipment and supplies	Shortage of equipment	Promote simple equipment
	Difficulty in maintenance	Low cost simulator training
	Acquisition and maintenance costs are high	—
Training and skills	A single training is insufficient	Blended learning
	Lack of practice opportunities	Remote supervision and quality feedback
	Lack of continuous feedback	Integrate FAST into medical schools and in-service training curricula
	Lack of standardized initial training	Waterfall training model
System and policy	Lack of standardized curriculum and clinical integration process	FAST should be clearly written into the national and regional trauma treatment guidelines and clinical pathways
	Insufficient policy support	A continuous quality control cycle and a regular skill retraining mechanism were established
Infrastructure	The electricity supply is erratic	Portable devices that use battery power
	Poor network connectivity	Use existing communication tools for remote support
	There is a shortage of basic consumables such as couplers	—

## 4 Discussion

This scoping review summarizes the current status of FAST in trauma care in resource-limited areas by systematically analyzing 29 studies. Evidence suggests that FAST has become a key decision-making tool for rapid diagnosis, triage and assessment in this setting. Its fast, portable, low-cost, and radiation-free features not only optimize the treatment process for individual patients but also have a strategic impact on improving the efficiency of health systems and rationally allocating scarce resources, underscoring its significant value in the field of global public health.

First, this review reveals the high adaptability and innovative potential that FAST has demonstrated in resource-limited environments. In settings where advanced imaging modalities like CT and MRI are scarce, FAST has emerged as a key bedside imaging tool for clinical decision-makers, with the potential to serve as a “critical screening tool” for frontline healthcare workers (29, 39). However, it is essential to clarify that its primary function is to detect intra-abdominal free fluid in trauma patients. Its role is that of an early screening method, not a comprehensive diagnostic assessment of injury, and it should never be regarded as a substitute for CT. This transformation was confirmed in a study conducted in India, where the application rate of FAST was as high as 99.2%, fully demonstrating that this technology has been deeply integrated into the daily trauma treatment process. Its application scenarios have also been extended from emergency departments to the most cutting-edge areas such as pre-hospital emergency care, primary clinics, and even field hospitals (20, 21, 24). Of particular significance is the effective “task transfer” that has enabled the FAST operators to expand from specialist doctors to non-physician clinical personnel (26, 29, 43), such as general practitioners and nurses. This provides a feasible solution to the core issue of shortage of professional manpower and lays the foundation for building a new public health trauma response system.

The core value of FAST lies in its systematic role as an “efficient diverter.” During the “golden period” of trauma treatment, its feature of rapid implementation at the bedside can significantly shorten the decision-making time and promote the evolution of clinical intervention towards greater precision and timeliness. A body of research has demonstrated that the inspection process has the capacity to enhance the allocation of key scarce resources, including but not limited to operating rooms and blood products. However, the diagnostic value of FAST is limited; it can only indicate the presence or absence of free fluid, whereas CT scans can clearly identify the specific location, severity, and nature of the injury. Meanwhile, FAST provides clinicians with timely decision-making confidence and enhances the efficiency of team collaboration. From a technical standpoint, the promotion of portable and durable convex array probe devices has the potential to substantially reduce the implementation threshold and cost (29). At the system level, explicitly incorporating FAST into the clinical pathways and protocols for trauma treatment, standardizing its application indications, interpretation standards, and corresponding clinical measures, serves as the institutional guarantee for promoting its regular and standardized use.

This review confirms that FAST has an excellent and consistently high specificity (94%–100%), which is the cornerstone of its public health utility (35, 38, 43). In situations where the risk of patient transport is high or CT is not available, its high specificity can effectively prevent unnecessary, costly and high-risk long-distance referral, and achieve safe and reasonable disposal de-escalation. Nevertheless, its sensitivity, particularly in detecting injuries to hollow organs, early solid injuries without free fluid, and retroperitoneal injuries, remains significantly limited. The improved techniques mentioned in the study, such as repeated ultrasound examination and the use of high-frequency probes, provide valuable practical implications for optimizing diagnostic performance based on existing technology. Critically, these technical enhancements do not alter the fundamental principle that FAST relies on the detection of free fluid as a positive finding. Therefore, they cannot overcome its inherent diagnostic blind spot for injuries not associated with free fluid. Even with future integration of AI-assisted image interpretation, this fundamental pathophysiological limitation cannot be overcome.

In resource-constrained settings, while FAST demonstrates significant clinical value, its inherent diagnostic limitations must be fully recognized. These limitations present a dual decision-making dilemma: for hemodynamically unstable patients, a positive result may support urgent intervention; yet for stable patients, relying solely on a positive result without CT validation may lead to unnecessary exploratory laparotomy. Conversely, a negative result cannot entirely rule out potential injury, necessitating repeated clinical and ultrasound assessments. Of particular significance is that non-surgical treatment strategies in modern trauma management—such as interventional embolization—rely heavily on the precise anatomical information provided by CT scans. FAST cannot furnish such support, effectively limiting the possibility of administering optimal treatment to suitable patients in resource-constrained settings. Therefore, FAST findings must be interpreted with precision within a comprehensive assessment framework encompassing injury mechanisms, the dynamic evolution of vital signs, and laboratory investigations. Implementing FAST in resource-constrained settings necessitates the simultaneous advancement of technology and the systematic establishment of clinical decision pathways and training frameworks that emphasize its role as a screening adjunct and clearly define its applicable boundaries. This approach maximizes its triage benefits while minimizing the risk of systemic misuse.

As demonstrated in the preceding literature, the successful and sustainable implementation of FAST promotion is not possible through a single training session. The key to achieving this objective is to establish a dynamic and continuous training and support system (28, 29, 34). Consequently, there is a necessity to integrate sustainable quality training cycles, peer learning groups, and institutionalised skills repetition training mechanisms into training. Training content must, in addition to mastering operational skills, encompass a profound understanding of FAST’s capability limitations—namely, that it serves merely as an extension and adjunct to the examination of trauma patients, rather than constituting a definitive diagnostic endpoint. However, this process faces multiple obstacles such as insufficient government support and systematic lack of resources. To this end, multi-level systematic strategies need to be adopted. For example, at the policy and institutional level, FAST should be integrated into national or regional trauma care guidelines to provide normative basis; In terms of capacity building, we should develop structured and sustainable training courses, train local ultrasound instructors, empower and support non-imaging specialists to implement FAST (24, 33). In terms of technological empowerment, innovative technologies such as smartphone adaptation probes and artificial intelligence assisted image reading are explored, and a remote guidance and quality feedback network is established to bridge the expert support gap caused by geographical distance and provide continuous training support for primary operators.

Nevertheless, the present study is not without its limitations. It is important to note that the present review excluded literature that extended ultrasonographic focused assessment of trauma (eFAST). The exclusion of eFAST may result in an inability on the part of this study to fully capture the evolution and full spectrum of ultrasonographic trauma assessment in resource-limited areas. However, the decision to exclude eFAST was made on the basis that it significantly differs from basic FAST with regard to procedural complexity, the depth of training required, and examination time (47, 48). The assessment of eFAST increases the difficulty of scanning and the complexity of interpretation (such as the thoracic region), and its skill acquisition and maintenance may pose a greater challenge for application in resource-limited Settings. In addition, part of the literature reported incomplete training details and implementation strategies, which limited the overall assessment of the strength of evidence and deeper comparative analysis to a certain extent.

## 5 Conclusion

In summary, FAST has significant application value and promotion potential in resource-limited areas, and its rapid, portable, low-cost, and radiation-free characteristics make it effective in enhancing the efficiency of initial trauma diagnosis, optimizing clinical decision-making processes, and potentially improving patient outcomes in the absence of advanced imaging equipment, which is an important role in improving the level of trauma care locally and globally. To maximize the benefits of FAST, continue to promote the development of portable, durable, low-cost ultrasound equipment and to develop and promote lower-cost, more adaptable training modalities. At the same time, we should actively integrate cutting-edge technologies to break through the existing limitations. In the future, the application of AI should not only be limited to assisting in image interpretation but can also be expanded to real-time operation guidance, scanning organ integrity prompts, and the construction of remote real-time quality control and consultation platforms so that grassroots ambulance crews in remote areas and disaster sites and those lacking professional imaging support will be able to provide instant professional support across geographic constraints. The promotion of FAST in LMICs and resource-poor areas is not only an innovative application of technology but also promotes the integration of multiple disciplines, including emergency medicine, public health, nursing, and health policy. FAST is expected to make a substantial contribution to improving the timeliness, effectiveness, and equity of trauma care and reducing mortality worldwide.

## Data Availability

The original contributions presented in the study are included in the article/supplementary material, further inquiries can be directed to the corresponding author.

## Footnotes

1
https://doi.org/10.17605/OSF.IO/2T7BK
